# Genetic and Environmental Influences on Caffeine Intake in Korean Twins

**DOI:** 10.1007/s10519-026-10276-y

**Published:** 2026-07-10

**Authors:** Minsu Cho, Jiyoung Youn, Moonil Kang, Kyunga Kim, Joohon Sung, Jung Eun Lee

**Affiliations:** 1 Department of Food and Nutrition, College of Human Ecology, Seoul National University, Seoul, 08826 Korea; 2 Department of Food and Nutrition, Chung-Ang University, Gyeonggi-do, 17546 Korea; 3 College of Pharmacy, Sookmyung Women’s University, Seoul, 04310 Korea; 4 Biomedical Statistics Center, Samsung Medical Center, Research Institute for Future Medicine, Seoul, 06351 Korea; 5 Department of Digital Health, Samsung Advanced Institute for Health Sciences & Technology, Sungkyunkwan University, Seoul, 06351 Korea; 6 Division of Genome and Health Big Data, Department of Public Health Sciences, Graduate School of Public Health, Seoul National University, Seoul, 08826 Korea; 7 Research Institute of Human Ecology, Seoul National University, Seoul, 08826 Korea

**Keywords:** Coffee drinking, Tea drinking, Caffeine intake, Heritability, Twins

## Abstract

Caffeine intake may be influenced by both genetic and environmental factors. This study aimed to examine the genetic contribution to variation in coffee and tea drinking, as well as total caffeine intake. A total of 1,106 Korean twins, comprising 456 monozygotic and 97 dizygotic twin pairs aged 30 years or older, from the Healthy Twin Study were included. Structural equation models were used to assess heritability estimates and their 95% confidence intervals (CIs). Heritability (95% CI) was estimated at 0.29 (0.21, 0.37) for coffee drinking, 0.11 (0.02, 0.20) for tea drinking, and 0.27 (0.16, 0.38) for total caffeine intake, with the remaining variance in each trait attributed to unique environmental factors. The unique environmental factors contributed more substantially to coffee drinking than genetic factors during early adulthood, while the genetic contribution to tea drinking remained consistently low across all ages. In conclusion, coffee drinking, tea drinking, and total caffeine intake were partly heritable, with unique environmental factors playing a predominant role.

**Supplementary Information:**

The online version contains supplementary material available at https://doi.org/10.1007/s10519-026-10276-y.

## Introduction

Coffee and tea are widely consumed beverages worldwide, serving as major dietary sources of caffeine (Fulgoni et al. 2015). Caffeine metabolism underlying enzymatic degradation, gastrointestinal absorption, hepatic biotransformation, and renal elimination may be determined by genetic factors. Genome-wide association studies (GWASs) have identified specific genetic loci linked to variation in caffeine intake and its physiological effects (Nehlig 2018).

Habitual coffee intake can be driven by the psychoactive properties of caffeine, which elevates alertness, improves mood, and enhances cognitive performance (van Dam et al. 2020). The structure of caffeine is similar to that of adenosine, allowing it to bind to adenosine receptors and inhibit adenosine-mediated effects, such as sedation and sleep promotion (Fredholm et al. 1999). However, at high doses or in sensitive individuals, caffeine can lead to anxiety, nervousness, and insomnia (van Dam et al. 2020). These interindividual differences may stem from genetic variation in caffeine metabolism and adenosine receptor genes. For example, carriers of the C allele for *CYP1A2* rs762551 exhibit slower caffeine metabolism than individuals with the AA genotype, appearing to consume less caffeine (Nehlig 2018). Twin studies have estimated the heritability of caffeine and coffee intake to range from 0.36 to 0.58 (Yang et al. 2010). Nevertheless, most of the existing evidence has been derived from Western populations, and the extent to which genetic and environmental factors explain caffeine and coffee intake in Asian populations remains uncertain.

Therefore, this study aimed to investigate the heritability of caffeine intake, including coffee and tea drinking, and total caffeine intake, in a Korean twin cohort.

## Methods

### Study Population

Data were obtained from the Healthy Twin Study, a family-based module of the Korean Genome and Epidemiology Study (KoGES) (Kim and Han 2017; Sung et al. 2006). This cohort was established to identify genetic variations associated with common complex diseases and traits. Participants were recruited between 2005 and 2013, including twins aged 30 years or older and their first-degree relatives aged 20 years or older.

Twin zygosity was determined using a validated questionnaire on physical similarity and the frequency with which others confused the twins, which was demonstrated to be as accurate as genotyping-based methods (Song et al. 2010). Monozygotic (MZ) twins were defined as pairs reporting mirror-like resemblance and being always or nearly always confused by teachers or friends. Dizygotic (DZ) twins were defined as pairs who did not report mirror-like resemblance and were seldom or never confused by others.

For the heritability analysis of coffee and tea drinking, the baseline study population included 3,500 participants. Participants were excluded if they met any of the following criteria: those who had a history of stroke, angina, myocardial infarction, or cancer (*n* = 452); those who had missing baseline food frequency questionnaires (FFQs) for coffee and tea intake (*n* = 60); or those who were non-twin family members (*n* = 1,882) (Figure S1). The final study population consisted of 1,106 twins, comprising 456 MZ twin pairs and 97 DZ twin pairs. For the heritability analysis of total caffeine intake, we additionally excluded 234 twin pairs (193 MZ and 41 DZ twin pairs) in which either twin had incomplete FFQ for other caffeine-containing food items. The higher proportion of MZ twins relative to DZ twins in our study population was attributable to the recruitment protocol of the Healthy Twin Study, which initially recruited same-sex twin pairs, thereby excluding opposite-sex DZ twin pairs (Gombojav et al. 2013; Sung et al. 2006). This was also consistent with the lower spontaneous DZ twinning rate than that of MZ twins in Korea prior to the widespread introduction of assisted reproductive technology in the early 1990s (Hur and Kwon 2005).

### Assessment of Caffeine Intake

As part of the Healthy Twin Study, caffeine intake was estimated using a 103-item semi-quantitative FFQ, the validity and reproducibility of which have been previously established (Ahn et al. 2007). Participants reported their intake of caffeine-containing foods and beverages over the past year, selecting from nine frequency categories ranging from almost never to three or more times per day. For coffee intake, the frequency of adding cream and sugar was also assessed. Portion sizes were recorded as half, equal to, or one-and-a-half or two times the standard serving size.

Coffee and tea drinking were classified as binary variables (current non-drinker or drinker) based on the intake levels reported by participants. Total daily caffeine intake was calculated as the sum of caffeine consumed from all relevant dietary sources, including coffee, tea, cake, chocolate pie, chocolate, flavored milk, ice cream, and carbonated beverage (coke). Caffeine content values for these items were determined using data from the National Institute of Food and Drug Safety Evaluation (Kim 2014), the United States Department of Agriculture food composition database (The United States Department of Agriculture), product nutrition labels, and direct inquiries with food manufacturers. Total caffeine intake was dichotomized into low and high intake based on the median value of the study population (55.55 mg/d).

### Assessment of Other Risk Factors

Trained research staff collected information on demographic and lifestyle factors (Kim and Han 2017). Variables included age, sex, weight, height, smoking status (never, former, and current), average number of cigarettes smoked per day, pack-years of smoking, alcohol drinking status (never, former, and current), alcohol drinking frequency and average serving size, marital status, education level, and monthly household income. We calculated body mass index (BMI) as weight in kilograms divided by height in meters squared (kg/m^2^).

### Statistical Analysis

To estimate the heritability, variance in traits was initially decomposed into additive genetic factors (A) and common environmental factors (C) shared between co-twins, and unique environmental factors (E) which include individual-specific environmental factors and measurement error. This decomposition was performed using a univariate structural equation model, which assumes that MZ twins share their entire genetic variation, whereas DZ twins share, on average, half of their segregating genes. By comparing cross-twin resemblance in caffeine intake between MZ and DZ twins, the relative contributions of genetic and environmental factors were quantified using the formula: h^2^ = σ_A_^2^/(σ_A_^2^ + σ_C_^2^ + σ_E_^2^), where σ_A_^2^ was the variance of additive genetic factors, σ_C_^2^ was the variance of common environmental factors, and σ_E_^2^ was the variance of unique environmental factors. Because the contribution of common environmental factors was negligible in the present study, the final analyses were conducted using the AE model based on the following formula: h^2^ = σ_A_^2^/(σ_A_^2^ + σ_E_^2^). Based on our sample size (456 MZ and 97 DZ twin pairs) and the observed heritability estimate (29% for coffee drinking), our study had 86.68% statistical power to detect a moderate common environmental component (30%) at a significance level of 0.05 (Visscher 2004). Additionally, to examine age-related variance in the genetic and environmental contributions to caffeine intake, we applied the AE(t) model, which estimates additive genetic and unique environmental variance components as smooth functions of age using B-spline basis functions, using the formula: h^2^(t) = σ_A_^2^(t)/[σ_A_^2^(t) + σ_E_^2^(t)]. In this analysis, we adjusted for sex (men and women). Participants younger than 30 years or older than 58 years (bottom and top 1%, respectively) were excluded to minimize potential bias from extreme ages.

Data cleaning and descriptive analyses were conducted using SAS 9.4 (SAS Institute Inc., Cary, NC, USA). Twin models were fitted using R 4.4.1 (R Core Team 2021) with the open-source packages mets (Scheike et al. 2014) and ACEt (He et al. 2017) for heritability estimation and OpenMx (Boker et al. 2011) for structural equation modeling. All models were adjusted for age (years, continuous) and sex (men and women), except for the age-specific heritability analyses which were adjusted for sex (men and women). The proportion of variance explained by the covariates was evaluated using Nagelkerke’s pseudo-R^2^ from logistic regression models adjusted for age and sex. To ensure the robustness of heritability estimates, we additionally adjusted for smoking status (never, < 10, 10 to < 25, and ≥ 25 pack-years), alcohol drinking (never, < 2, 2 to < 8, and ≥ 8 cups/week), BMI (< 23, 23 to < 25, and ≥ 25 kg/m^2^), education level (middle school or below, high school, and some college or above), household income (< 2, 2 to < 4, and ≥ 4 million Korean won/month), marital status (single, separated, divorced, or widowed and married or cohabiting), and total energy intake (kcal/d, continuous). These additional covariates were incorporated after excluding participants with missing data on total energy intake (*n* = 28 for coffee and tea drinking and *n* = 14 for total caffeine intake). Additionally, we also estimated heritability using continuous measures of coffee intake (cups/d), tea intake (cups/d), and total caffeine intake (mg/d). Further analyses were conducted using alternative categorical definitions for current coffee drinking (non-drinker or ≥ 1 cup/d) and current tea drinking (non-drinker or ≥ 0.5 cup/d). Heritability estimates were considered statistically significant if their 95% confidence intervals (CIs) did not include zero.

## Results

Baseline characteristics by zygosity are presented in Table 1. A total of 1106 twins were included, consisting of 456 MZ twin pairs and 97 DZ twin pairs. The overall mean age of participants was 38.64 ± 7.47 years, with mean ages of 38.49 ± 7.44 years for MZ twins and 39.32 ± 7.55 years for DZ twins. Compared with MZ twins, DZ twins had a higher proportion of participants aged ≥ 50 years (*p* = 0.039) and men (*p* = 0.013), and had lower education levels (*p* = 0.017). Otherwise, no statistically significant differences were observed in BMI, smoking status, alcohol drinking, marital status, or total energy intake (all *p* > 0.05). Regarding caffeine intake, 88% of participants were current coffee drinkers, and 78% were current tea drinkers. The mean daily caffeine intake from all dietary sources was 64.85 ± 52.11 mg, with MZ twins consuming 64.14 ± 52.98 mg and DZ twins consuming 68.17 ± 47.88 mg.

**Table 1:** Baseline characteristics of the study population

	Total	MZ	DZ	*p* value^a^
No. of participants	1,106	912	194	
Age (years)	38.64 (7.47)	38.49 (7.44)	39.32 (7.55)	0.172
< 30	44 (4.0)	42 (4.6)	2 (1.0)	0.039
30 to < 40	658 (59.5)	540 (59.2)	118 (60.8)	
40 to < 50	284 (25.7)	238 (26.1)	46 (23.7)	
≥ 50	120 (10.8)	92 (10.1)	28 (14.4)	
Sex				
Men	426 (38.5)	336 (36.8)	90 (46.4)	0.013
Women	680 (61.5)	576 (63.2)	104 (53.6)	
Body mass index (kg/m^2^)	23.05 (2.98)	23.01 (2.95)	23.21 (3.12)	0.461
Smoking status (pack-years)^b^	4.08 (8.20)	3.79 (7.60)	5.47 (10.51)	0.838
Never	719 (65.0)	602 (66.0)	117 (60.3)	0.303
Former	129 (11.7)	104 (11.4)	25 (12.9)	
Current	257 (23.2)	205 (22.5)	52 (26.8)	
Alcohol drinking (cups/week)^b^	6.42 (11.49)	6.52 (11.76)	5.97 (10.20)	0.799
Never	252 (22.8)	207 (22.7)	45 (23.2)	0.530
Former	97 (8.8)	84 (9.2)	13 (6.7)	
Current	756 (68.4)	620 (68.0)	136 (70.1)	
Education level^b^				
Middle school or below	118 (10.7)	90 (9.9)	28 (14.4)	0.017
High school	506 (45.8)	408 (44.7)	98 (50.5)	
Some college or above	481 (43.5)	413 (45.3)	68 (35.1)	
Marital status^b^				
Single, separated, divorced, or widowed	303 (27.4)	248 (27.2)	55 (28.4)	0.749
Married or cohabiting	802 (72.5)	663 (72.7)	139 (71.6)	
Coffee intake (cups/d)	1.06 (1.01)	1.04 (1.00)	1.17 (1.05)	0.067
Non-current	130 (11.8)	112 (12.3)	18 (9.3)	0.238
Current	976 (88.2)	800 (87.7)	176 (90.7)	
Tea intake (cups/d)	0.51 (0.79)	0.52 (0.82)	0.48 (0.67)	0.977
Non-current	246 (22.2)	200 (21.9)	46 (23.7)	0.588
Current	860 (77.8)	712 (78.1)	148 (76.3)	
Total caffeine intake (mg/d)^b^	64.85 (52.11)	64.14 (52.98)	68.17 (47.88)	0.123
Total energy intake (kcal/d)^b^	1,879 (827.2)	1,863 (814.0)	1,951 (884.0)	0.061

Abbreviation:* MZ* monozygotic twins,* DZ* dizygotic twins

Continuous variables are presented as mean (standard deviation), and categorical variables are presented as no. (%).

^a^ Continuous variables were compared using the Wilcoxon rank-sum test, and categorical variables were compared using the chi-square tests.

^b^ The total number of participants was not equal because of missing values.

The estimated variance components for caffeine intake are summarized in Table 2. Intraclass correlation for coffee drinking was higher among MZ twins than among DZ twins, suggesting significant heritability. Genetic factors accounted for 0.29 (95% CI 0.21, 0.37) of the variance in coffee drinking, with unique environmental factors explaining the majority (0.71; 95% CI 0.63, 0.79). Tea drinking showed minimal heritability (0.11; 95% CI 0.02, 0.20), with overlapping intraclass correlations observed between MZ and DZ twins. Unique environmental factors accounted for the largest proportion of the variance in tea drinking (0.89; 95% CI 0.80, 0.98). For total caffeine intake, about three-quarters of the variance was attributable to unique environmental factors (0.73; 95% CI 0.62, 0.84), and genetic factors explained the remainder (0.27; 95% CI 0.16, 0.38). Covariates including age and sex explained a small proportion of the total variance in caffeine intake (Nagelkerke R² = 0.004 for coffee drinking, 0.002 for tea drinking, and 0.006 for total caffeine intake). Goodness-of-fit statistics for the univariate twin models are presented in Table S1. The heritability estimates remained largely unchanged after additional adjustments for smoking status, alcohol drinking, BMI, education level, household income, marital status, and total energy intake (Table S2). When we examined the heritability of caffeine intake using continuous variables, genetic factors explained 0.27 (0.19, 0.35) of the variance in coffee intake, 0.10 (0.01, 0.18) in tea intake, and 0.17 (0.06, 0.28) in total caffeine intake (Table S3). Using alternative classification methods, the heritability estimates (95% CIs) were 0.50 (0.41, 0.59) for coffee drinking (non-drinker or ≥ 1 cup/d) and 0.23 (0.09, 0.37) for tea drinking (non-drinker or ≥ 0.5 cup/d) (Table S4).

**Table 2:** Intraclass correlations and heritability estimates with 95% confidence intervals (CIs) of caffeine intake

	Intraclass correlation		Additive genes	Unique environment
	MZ	DZ		
Coffee drinking	0.29 (0.21, 0.37)	0.15 (0.10, 0.19)	0.29 (0.21, 0.37)	0.71 (0.63, 0.79)
Tea drinking	0.11 (0.02, 0.20)	0.05 (0.01, 0.10)	0.11 (0.02, 0.20)	0.89 (0.80, 0.98)
Total caffeine intake	0.27 (0.15, 0.37)	0.13 (0.08, 0.19)	0.27 (0.16, 0.38)	0.73 (0.62, 0.84)

Abbreviation: *MZ* monozygotic twins, *DZ* dizygotic twins.

Caffeine intake behaviors were classified as binary variables (current non-drinker or drinker for coffee and tea drinking, and low or high for total caffeine intake).

Estimates were derived from the AE twin model adjusted for age (years, continuous) and sex (men and women).

**Fig. 1:**
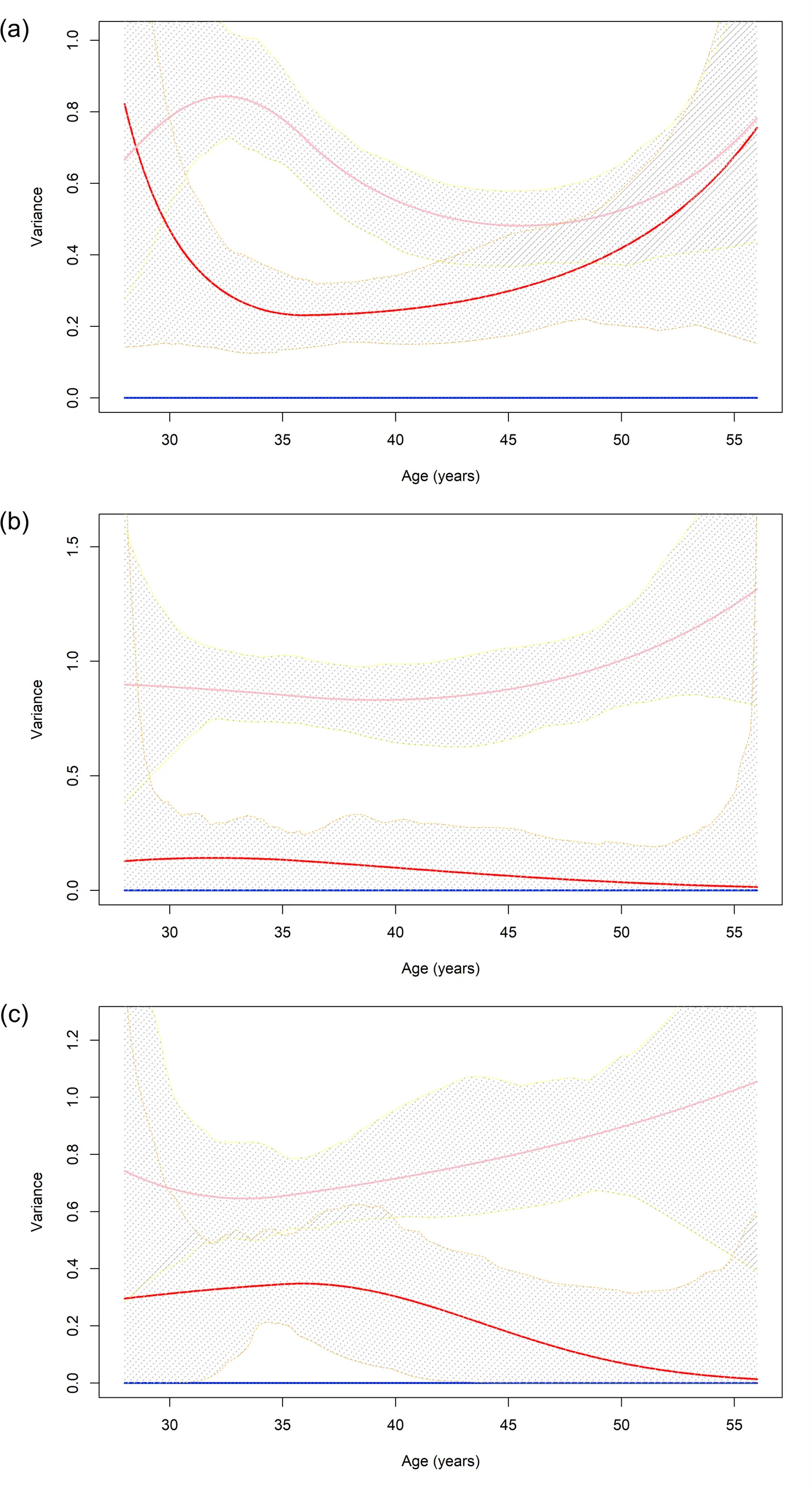
Variance curves of genetic and environmental factors for caffeine intake. The red, blue, and pink lines represent genetic, common environmental, and unique environmental variance, respectively. Shaded areas indicate the 95% confidence intervals. Variance curves across age are shown for (**a**) coffee drinking, (**b**) tea drinking, and (**c**) total caffeine intake. Caffeine intake behaviors were classified as binary variables (current non-drinker or drinker for coffee and tea drinking, and low or high for total caffeine intake).

Figure 1 illustrates the age-specific estimates of genetic and environmental variance components for caffeine intake. For coffee drinking, the genetic variance was relatively higher in early adulthood, declined until approximately age 35, and then gradually increased in later adulthood (Fig. 1a). Tea drinking showed consistently low genetic variance across all ages, whereas the unique environmental variance was higher among participants older than 50 years (Fig. 1b). For total caffeine intake, the unique environmental variance exceeded the genetic variance, particularly after mid-adulthood (Fig. 1c). Goodness-of-fit statistics for the age-specific univariate twin models are summarized in Table S5.

## Discussion

This study examined the genetic and environmental contributions to caffeine intake in a Korean twin cohort. We found significant heritability for coffee drinking (29%), tea drinking (11%), and total caffeine intake (27%), with unique environmental factors accounting for the remaining variance. These results remained consistent when analyzed as continuous variables.

There were significant genetic contributions to coffee drinking and total caffeine intake, and unique environmental factors explained the remaining variance. These findings align with previous twin studies showing that caffeine-related traits are subject to both genetic and unique environmental factors. For example, Finnish twins exhibited substantial heritability for coffee intake (0.56 in 1975 and 0.45 in 1981), with comparable unique environmental influences (Laitala et al. 2008). Similarly, a Dutch twin study attributed 39% of the variance in coffee intake to genetic factors, while common and unique environmental factors contributed 21% and 40%, respectively (Vink et al. 2009). Parallel findings were reported from twin cohorts in Norway (45% genetic and 55% unique environmental factors) (Czajkowski et al. 2021) and the United States (43% genetic and 57% unique environmental factors) (Kendler and Prescott 1999).

Though the heritability estimates for coffee drinking and total caffeine intake in this study were overall consistent with those reported in Western twin studies, they appeared relatively lower in magnitude. This may reflect ethnic differences in both cultural and genetic factors. For instance, the average caffeine intake in our study population (65 mg/d) was substantially lower than in the United States (186 mg/d), which may reduce phenotypic variability and the detectable genetic contribution (Fulgoni et al. 2015). Importantly, when coffee drinking was classified as ≥ 1 cup/d versus non-drinker, heritability was estimated at 0.50, closely aligning with the heritability estimates reported in Western populations. This indicates that genetic influences on coffee drinking may be more pronounced at higher levels of intake. Moreover, the frequency of the *CYP1A2* rs762551 A allele, linked to faster caffeine metabolism, is lower in East Asians (0.57) than in Europeans (0.70) (National Library of Medicine), potentially contributing to the observed difference in heritability. Interindividual differences in caffeine intake likely stem from genetic predispositions related to caffeine metabolism. Variants in genes regulating caffeine pharmacokinetics (e.g., *ABCG2*, *AHR*, *POR*, and *CYP1A2*) and pharmacodynamics (e.g., *BDNF* and *SLC6A4*) were shown to influence caffeine clearance rates and physiological properties (Cornelis et al. 2015). Individuals with faster caffeine metabolism may develop greater caffeine tolerance, thereby increasing their likelihood of caffeine use.

Compared to coffee drinking, tea drinking exhibited relatively lower heritability, suggesting a predominantly environmental basis. This is consistent with an Australian twin study that estimated heritability at 0.51 for coffee intake and 0.26 for tea intake (Luciano et al. 2005). Among Chinese male twins, tea intake was primarily influenced by common (64%) and unique environmental factors (20%), with genetic contributions limited to 17% (Dongmeng et al. 2022). The lower heritability of tea drinking may be partly explained by its high susceptibility to environmental factors, such as cultural practices and lifestyle choices, and its lower caffeine content relative to coffee (18.37 mg/g for coffee and 0.14 mg/g for tea in this study).

We further identified age-specific variations in the genetic and environmental variance of coffee and tea drinking. Genetic contributions to coffee drinking were more pronounced in early adulthood than in mid-adulthood, likely due to inherited traits including genetic polymorphisms related to caffeine metabolism. However, as individuals age, environmental factors such as workplace norms and social networks appeared to exert a greater influence. Our findings are similar to those from a Finnish twin study reporting minimal genetic variance of coffee intake during middle age, when environmental factors may strongly shape coffee intake behaviors (Laitala et al. 2008). For tea drinking, we observed that environmental variance was higher in later life than midlife, while maintaining consistently minimal genetic variance across ages. It is possible that personal factors, such as health conditions, economic status, and lifestyle habits, may be major determinants of tea intake compared to genetic factors. Nevertheless, given the wide overlapping confidence intervals across age groups, these age-specific patterns should be interpreted with caution. Future longitudinal studies are required to further elucidate these age-related variations.

There are limitations in this study to acknowledge. First, though we used the AE model as the most parsimonious model, the possibility of common environmental contributions cannot be entirely ruled out. Nonetheless, our study had sufficient statistical power to detect a moderate common environmental effect. Further studies on the heritability of caffeine intake are warranted. Second, coffee intake was assessed using FFQ, which is prone to measurement error. Notably, since unique environmental variance includes measurement error, it should be noted when interpreting the estimates of unique environmental factors. However, prior research using KoGES data has shown a 71% agreement between the FFQ and two-day 24-hour recalls in identifying coffee non-drinkers (Kim et al. 2020). Third, other dietary caffeine sources, such as energy drinks, were not included in our assessment, but energy drinks contribute minimally to total caffeine intake among Korean adults (0.80% in those aged 19 to 29 years, 0.44% in those aged 30 to 49 years, and 0.22% in those aged 50 to 64 years) (Lim et al. 2015). Finally, despite careful adjustments for potential confounding variables, residual confounding cannot be entirely ruled out.

This study also has strengths. First, to our knowledge, this is the first study that examined the heritability of coffee and total caffeine intake among East Asians. Second, the inclusion of twins across a wide age range enabled the investigation of the genetic architecture across ages.

In conclusion, significant genetic contributions to coffee drinking, tea drinking, and total caffeine intake were identified in Korean twins, with unique environmental factors accounting for the majority of the variance. Future longitudinal research considering genetic, cultural, and dietary variations in caffeine intake is necessary to further clarify these findings, particularly in Asian populations.

## Supplementary Information

Below is the link to the electronic supplementary material.


Supplementary Material 1


## Data Availability

Data are available on request due to privacy and ethical restrictions.
