# Supplementary material for: Genetic and Environmental Influences on Caffeine Intake in Korean Twins
Source: Behav Genet. 2026 Jul 10;56(5):236–43. doi: 10.1007/s10519-026-10276-y (PMC13529835; doi:10.1007/s10519-026-10276-y)
Supplement: Supplementary file 1 [file 10519_2026_10276_MOESM1_ESM.docx]

**Supplementary materials**

Baseline participants (n = 3,500)

Excluded (n = 2,394)

Participants who

- had a history of stroke, angina, myocardial infarction, or cancer (n = 452)

- had missing baseline food frequency questionnaires (FFQs) for coffee and tea (n = 60)

- were non-twin family members (n = 1,882)

Final study population (n = 1,106)

(n = 912 monozygotic twins and n = 194 dizygotic twins)

**Figure S1.** Flow diagram of the study population included in the analysis

**Table S1.** Goodness-of-fit statistics for univariate structural equation models of caffeine intake

|  | **–2 ln *L*** | ***df*** | **AIC** | **BIC** |
| --- | --- | --- | --- | --- |
| **Coffee drinking** |  |  |  |  |
| ACE | 585.91 | 6 | 597.91 | 623.81 |
| AE | 585.91 | 5 | 595.91 | 617.49 |
| CE | 589.33 | 5 | 599.33 | 620.91 |
| E | 629.92 | 4 | 637.92 | 655.18 |
| **Tea drinking** |  |  |  |  |
| ACE | 1,190.11 | 6 | 1,202.11 | 1,228.00 |
| AE | 1,190.11 | 5 | 1,200.11 | 1,221.68 |
| CE | 1,192.20 | 5 | 1,202.20 | 1,223.78 |
| E | 1,195.57 | 4 | 1,203.57 | 1,220.84 |
| **Total caffeine intake** |  |  |  |  |
| ACE | 903.51 | 6 | 915.51 | 938.10 |
| AE | 903.51 | 5 | 913.51 | 932.33 |
| CE | 906.97 | 5 | 916.97 | 935.80 |
| E | 923.68 | 4 | 931.68 | 946.74 |
| Abbreviations: –2 ln *L*, twice the negative log-likelihood; *df*, degrees of freedom; AIC, Akaike information criterion; BIC, Bayesian information criterion; A, additive genetic factors; C, common environmental factors; E, unique environmental factors.  Caffeine intake behaviors were classified as binary variables (non-drinker or drinker for coffee and tea drinking, and low or high for total caffeine intake).  Models were adjusted for age (years, continuous) and sex (men and women). | | | | |

**Table S2.** Intraclass correlations and heritability estimates with 95% confidence intervals (CIs) of caffeine intake with additional adjustment

|  | **Intraclass correlation** | | **Additive genes** | **Unique environment** |
| --- | --- | --- | --- | --- |
|  | **MZ** | **DZ** |  |  |
| Coffee drinking | 0.28 (0.19, 0.38) | 0.14 (0.09, 0.19) | 0.28 (0.19, 0.38) | 0.72 (0.62, 0.81) |
| Tea drinking | 0.10 (0.00, 0.19) | 0.05 (0.00, 0.10) | 0.10 (0.00, 0.19) | 0.90 (0.81, 1.00) |
| Total caffeine intake | 0.25 (0.13, 0.36) | 0.12 (0.06, 0.18) | 0.25 (0.13, 0.37) | 0.75 (0.63, 0.87) |
| Abbreviations: MZ, monozygotic twins; DZ, dizygotic twins.  Caffeine intake behaviors were classified as binary variables (non-drinker or drinker for coffee and tea drinking, and low or high for total caffeine intake).  Estimates were derived from the AE twin model adjusted for age (years, continuous), sex (men and women), smoking status (never, < 10, 10 to < 25, and ≥ 25 pack-years), alcohol drinking (never, < 2, 2 to < 8, and ≥ 8 cups/week), body mass index (< 23, 23 to < 25, and ≥ 25 kg/m^2^), education level (middle school or below, high school, and some college or above), household income (< 2, 2 to < 4, and ≥ 4 million Korean won/month), marital status (single, separated, divorced, or widowed and married or cohabiting), and total energy intake (kcal/d, continuous). | | | | |

**Table S3.** Intraclass correlations and heritability estimates with 95% confidence intervals (CIs) of caffeine intake as continuous variables

|  | **Intraclass correlation** | | **Additive genes** | **Unique environment** |
| --- | --- | --- | --- | --- |
|  | **MZ** | **DZ** |  |  |
| Coffee intake (cups/d) | 0.27 (0.18, 0.35) | 0.13 (0.09, 0.18) | 0.27 (0.19, 0.35) | 0.73 (0.65, 0.81) |
| Tea intake (cups/d) | 0.10 (0.01, 0.18) | 0.05 (0.01, 0.09) | 0.10 (0.01, 0.18) | 0.90 (0.82, 0.99) |
| Total caffeine intake (mg/d) | 0.17 (0.06, 0.28) | 0.08 (0.03, 0.14) | 0.17 (0.06, 0.28) | 0.83 (0.72, 0.94) |
| Abbreviations: MZ, monozygotic twins; DZ, dizygotic twins.  Estimates were derived from the AE twin model adjusted for age (years, continuous) and sex (men and women). | | | | |

**Table S4.** Intraclass correlations and heritability estimates with 95% confidence intervals (CIs) of coffee and tea drinking based on alternative classification methods

|  | **Intraclass correlation** | | **Additive genes** | **Unique environment** |
| --- | --- | --- | --- | --- |
|  | **MZ** | **DZ** |  |  |
| Coffee drinking | 0.50 (0.41, 0.58) | 0.25 (0.21, 0.29) | 0.50 (0.41, 0.59) | 0.50 (0.41, 0.59) |
| Tea drinking | 0.23 (0.09, 0.37) | 0.12 (0.05, 0.19) | 0.23 (0.09, 0.37) | 0.77 (0.63, 0.91) |
| Abbreviations: MZ, monozygotic twins; DZ, dizygotic twins.  Caffeine intake behaviors were classified as binary variables (non-drinker or ≥ 1 cup/d for coffee drinking and non-drinker or ≥ 0.5 cup/d for tea drinking).  Estimates were derived from the AE twin model adjusted for age (years, continuous) and sex (men and women). | | | | |

**Table S5.** Goodness-of-fit statistics for age-specific univariate structural equation models of caffeine intake

|  | **–2 ln *L*** | ***k*** | **AIC** | **BIC** |
| --- | --- | --- | --- | --- |
| **Coffee drinking** |  |  |  |  |
| ACE | 970.82 | 14 | 998.82 | 1,068.53 |
| AE | 1,006.38 | 9 | 1,024.38 | 1,069.19 |
| CE | 991.02 | 11 | 1,013.02 | 1,067.79 |
| E | 1,057.01 | 6 | 1,069.01 | 1,098.88 |
| **Tea drinking** |  |  |  |  |
| ACE | 1,055.49 | 14 | 1,083.49 | 1,153.20 |
| AE | 1,061.70 | 9 | 1,079.70 | 1,124.51 |
| CE | 1,060.61 | 11 | 1,082.61 | 1,137.38 |
| E | 1,068.01 | 6 | 1,080.01 | 1,109.88 |
| **Total caffeine intake** |  |  |  |  |
| ACE | 585.73 | 14 | 613.67 | 679.39 |
| AE | 588.32 | 9 | 606.33 | 648.58 |
| CE | 589.71 | 11 | 611.68 | 663.32 |
| E | 611.99 | 6 | 623.98 | 652.15 |
| Abbreviations: –2 ln *L*, twice the negative log-likelihood; *k*, number of parameters; AIC, Akaike information criterion; BIC, Bayesian information criterion; A, additive genetic factors; C, common environmental factors; E, unique environmental factors.  Caffeine intake behaviors were classified as binary variables (non-drinker or drinker for coffee and tea drinking, and low or high for total caffeine intake).  Models were adjusted for sex (men and women). | | | | |
